# Comparative evaluation of tensile bond strengths of total-etch adhesives and self-etch adhesives with single and multiple consecutive applications: An *in vitro* study

**DOI:** 10.4103/0972-0707.55618

**Published:** 2009

**Authors:** Deepthi Mandava, Ajitha P, L Lakshmi Narayanan

**Affiliations:** Department of Conservative Dentistry and Endodontics, Narayana Dental College, Nellore Dist., Andhra Pradesh, India; 1 Department of Conservative Dentistry and Endodontics, Adhiparashakthi Dental College, Melmaruvathur Village, India; 2 Department of Conservative Dentistry and Endodontics, S.R.M. Dental College, Kattankulathur, Kancheepuram Dt., Tamil Nadu, India

**Keywords:** Multiple consecutive applications, resin-dentin bond, self-etch adhesives, tensile bond strength, total-etch adhesives

## Abstract

**Aim:**

This study evaluates the effect of single and multiple consecutive applications of adhesives on the tensile bond strength. The currently available adhesives follow either the total-etch or the self-etch concept. However, in both techniques the uniformity and thickness of the adhesive layer plays a significant role in the development of a good bond.

**Materials and Methods:**

Sixty composite-dentin bonded specimens were prepared using a total-etch adhesive (Gluma) and another 60 using a self-etch adhesive (AdheSE). Each group was further divided into six subgroups based on the number of applications, i.e., single application and multiple (2, 3, 4, 6, and 8) applications. The tensile bond strength was tested with the Instron universal testing machine. The values were analyzed with one-way ANOVA and multiple range tests by Tukey's HSD procedure to identify those subgroups that had significantly higher bond strength.

**Results:**

The results indicate that with total-etch adhesive the bond strength increases significantly as the number of applications are increased from one to two or from two to three”, for self-etch adhesive the bond strength obtained with two applications is significantly higher than that with one application. However, for both adhesive systems, there was a decrease in the tensile bond strength values with further applications.

**Conclusion:**

We conclude that, in the clinical setting, the application of multiple coats of total etch adhesive improves bonding.

## INTRODUCTION

Concepts in restorative dentistry have been continually changing during the past four decades, and adhesive dentistry has steadily gained importance. The trend in favor of adhesive dentistry started in the mid 1960s with the advent of the first commercial restorative resin composites.[1] Modern adhesive dentistry offers significant advantages; for example, it allows conservation of hard tissue and makes possible effective and efficient restoration. The goal in adhesive dentistry is to achieve an adequately strong bonding of the restorative resin to the tooth structure so that there is optimum retention, minimal microleakage and, hence, better color stability and clinical longevity of the restoration.

Originally, acid conditioning and adhesive application was used only on enamel surfaces, but now acceptable dentin bonding is possible; it depends upon the formation of a hybrid layer that is optimally infiltrated with adhesive resin. The formation of hybridized dentin is greatly dependent upon the permeability of the dentin substrate and the diffusion potential of the adhesive monomer.[2]

Current strategies in adhesive dentistry involve two methods: the total-etch bonding technique is characterized by the complexity of its components and of the bonding procedure. Self-etching systems follow a trend towards simplification.[3] Simultaneous etching of enamel and dentin is the basis for the total-etch technique which leads to hybridization at resin-dentine interface by a molecular level mixture of adhesive polymers and dentinal hard tissues.

Over priming is as detrimental as under priming of dentin. The self-etching primer and adhesives were developed in order to avoid the adverse effects of over etching and under/over priming. One advantage with the use of self-etch adhesives is that prior removal of the smear layer and smear plugs is not required as these systems are capable of etching the tooth surface, while simultaneously preparing it for adhesion.[3]

The consecutive coating method, with multiple applications of resin, is a simple technique that improves the quality of resin–dentin bonds. Many studies have demonstrated the effectiveness of multiple applications of self-etch adhesives in increasing bond strength, but there is very limited information available on the effect of multiple consecutive coats of total-etch adhesives.[4]

We hypothesized that multiple consecutive coatings of adhesives will increase resin infiltration into the etched, moist dentin and thereby increase bond strength. The aim of this study was to evaluate the effect of single and multiple consecutive applications of total-etch and self-etch adhesives on dentin bond strength.

## MATERIALS AND METHODS

A total of 120 noncarious, freshly extracted human premolars stored in normal saline were used for the study. The teeth were ultrasonically cleaned and mounted in a phenolic ring using self-cure acrylic resin. The occlusal surface was ground in a water-cooled model trimmer until all enamel was removed (except at the periphery). This procedure resulted in exposure of a flat dentin surface located slightly apical to the mid-coronal level and oriented perpendicular to the long axis of the mold. The dentin was hand-polished to 600-grit on a series of wet silicon carbide abrasive papers. The surface was examined after polishing to ensure that the orientation was not altered.

The specimens were randomly divided into two groups of 60 teeth each and, further, into six subgroups with 10 teeth in each. Group I was treated with a total-etch adhesive (Gluma, Heraeus Kulzer, Germany 010066) and group II with a self-etch adhesive (AdheSE, Ivoclar Vivadent, Amherst, NY USA, F 25882). The compositions of the two adhesives are shown in Table 1.

**Table 1:** Composition of adhesives

Group	Bonding agent		Composition
I	Gluma (Total-etch)	Adhesive	Etchant
		HEMA	35% H3P04
		4 META	
		Methacrylate	
		polycarboxylic	
		gluteraldehyde	
		Ethanol and water	
II	AdheSE	Primer	Adhesive
	(Self-etch)	bis-acrylamide	HEMA
		Phosphoric acid	Dimethacrylate
		Initiators	Silicon dioxide
		Stabilizers	Initiators
		Water	Stabilizers

### Group I

The prepared dentin surfaces were acid conditioned for 15 s (total-etch) and subsequently washed thoroughly for 10 s using a water spray. Excess water was blot-dried with a cotton pellet, leaving the surface visibly moist. For single applications of the Gluma adhesive system, the bonding adhesive was applied to the entire dentin surface without agitation and allowed to dwell undisturbed. The solvent was then gently evaporated to form a slightly shiny adhesive film; it was light cured for 20 s using a light-curing unit. For multiple consecutive applications (2, 3, 4, 6, and 8 applications), the adhesive application and solvent evaporation steps were done repeatedly, with light curing done only after all layers had been applied.[4] After the final application and solvent evaporation, the adhesive layer was light cured for 20 s with a light-curing unit (Caulk Dentsply, USA).

### Group II

For AdheSE, the primer was applied on prepared dentin surfaces for 30 s and air-dried according to the manufacturer's instructions. Subsequently, the adhesive was gently applied all over the surface (as described above) to form a shiny adhesive film and was light cured for 10 s. Multiple consecutive coatings were applied in the same manner as described in the case of the Gluma adhesive system. After the final application and solvent evaporation, the adhesive layer was light cured for 10 s with a light-curing unit.

At the completion of the bonding procedure, composite (Charisma, Heraeus Kulzer) was loaded into a 4-mm capsule and applied to the treated dentin surface. Excess material was removed from the periphery with an explorer. The composite was cured for 20 s from each of four directions at a 45° angle to the bonding interface.

For tensile bond testing, the specimens were mounted in an Instron universal testing machine at a crosshead speed of 1 mm/min. The maximum load (N) applied on each specimen to fracture was divided by the cross-sectional area of the bonded composites to determine the tensile bond strength in MPa. Data were subjected to one-way analysis of variance (ANOVA) (*P*<0.0001) and multiple range test by Tukey-HSD procedure to identify the significant groups (*P*<0.05).

## RESULTS

The mean bond strengths for each adhesive system, as a function of the number of applications of the adhesive, are shown in Table 2. The bond strength of Gluma shows statistically significant increase as the number of coatings increase from one to two (15.97 ± 0.40 and 17.49 ± 0.39 MPa, respectively) and from two to three (17.49 ± 0.39 and 22.29 ± 0.70 MPa, respectively), followed by decrease in bond strength with successive coatings. Similarly, in the case of AdheSE, bond strength with two consecutive coatings (16.30 ± 0.15 MPa) was significantly higher than that with a single coating (10.51 ± 0.56 to), but application of further coatings caused a decrease in bond strength. For all subgroups, mean values of bond strength in group I (total-etch) is significantly higher than the mean values in group II (self-etch) (*P* < 0.0001).

**Table 2:** Mean bond strength of adhesives as a function of the number of applications

Subgroup (No. of coats)	Group I (Total–etch)	Group II (Self–etch)
	Mean ± S.D	Mean ± S.D
1	15.97 ± 0.40*	10.51 ± 0.56*
2	17.49 ± 0.39*	16.30 ± 0.15*
3	22.29 ± 0.70*	15.42 ± 0.19*
4	15.64 ± 0.31*	14.29 ± 0.23*
6	15.23 ± 0.47*	10.66 ± 0.54*
8	15.85 ± 0.19*	10.00 ± 0.14*

*Statistically significant groups. P value < 0.0001

Comparison of mean values of bond strength between different subgroups for group I and group II are shown in Table 3. For group I the mean value in subgroup 3 (22.29 ± 0.70) is significantly higher than the mean values in all the other subgroups (*P* < 0.05). Also the mean value in subgroup 2 is significantly higher than the mean value in subgroup 1, 4, 6, and 8 (*P* < 0.05). The other differences were not statistically significant (*P* > 0.05). For group II, the mean value in subgroup 2 (16.30 ± 0.15 MPa) is significantly higher than the mean values in subgroup 1,3,4,6 and 8 (*P* < 0.05) and also the mean value in subgroup 3 and subgroup 4 is significantly higher than the mean value in sibgroup 1,6,8 (*P* < 0.05). No other contrasts are statistically significant (*P* > 0.05).

**Table 3:** Comparison of mean values between subgroups of group I and group II

Group	Subgroup	Mean ± SD
I	1	15.97 ± 0.40
	2	17.49 ± 0.39*
	3	22.29 ± 0.70*
	4	15.64 ± 0.31
	6	15.23 ± 0.47
	8	15.85 ± 0.19
II	1	10.51 ± 0.56
	2	16.30 ± 0.51*
	3	15.42 ± 0.19*
	4	14.29 ± 0.23*
	6	10.66 ± 0.54
	8	10.00 ± 0.14

*Statistically significant groups. P value < 0.05

The maximum bond strength in this study was recorded in group 1, with three coatings of Gluma (22.29 ± 0.70 MPa); in group 2, the maximum bond strength was seen with two coatings of AdheSE (16.30 ± 0.15).

## DISCUSSION

Adhesion is defined as the mechanism that bonds two materials in intimate contact across an interface.[5] The key element for adhesion is the intimacy of the bond that develops between the adhesive and the substrate.[2] While effective adhesion to enamel is achieved with relative ease, adhesion to dentin poses a difficult challenge.[6] This is partly due to the biological characteristics of dentin, namely its high organic content, its tubular structure, and the presence of the dentin smear layer that forms immediately after cavity preparation.[7] This smear layer prevents the adhesive from interacting directly with dentinal tissue.[8] Unless the smear layer is modified or removed, neither hybridization nor resin tag formation can occur.

It is well accepted that bond strength is affected by the extent of resin infiltration into the exposed collagen network.[8] Different techniques of acid conditioning, application of adhesive resin, and evaporation of solvents can change the amount of resin uptake and the resulting bond strength.[4]

It is accepted that acid etching is essential for good dentin bonding; the question is whether the etched dentin must be dehydrated, rehydrated, or something in between to achieve the best results. Collagen fibrils collapse when totally dehydrated and this impairs the formation of a hybrid layer. To overcome this problem, water-based dentin bonding agents were developed that would rehydrate the collagen. The primer takes less time to improve permeability of moist dentine as compared to rehydrated demineralized dentine.

Over-wetting the dentin causes swelling of the collagen network (i.e., there is increase in the diameter and length of the collagen fibrils); this results in decrease in the width of the perifibrillar spaces, thereby reducing the diffusion of monomers. What we need is a system that can compete with moisture and replace it; it should be capable of taking the monomer along with it. After it has replaced the moisture it must evaporate away. This is possible with high-vapor-pressure solvents such as acetone and ethanol. Water-based adhesives help in rewetting, whereas organic solvent–based adhesives offer better infiltration. Therefore, the inclusion of both (a high-vapor-pressure solvent and water) may be fundamental for achieving adequate infiltration, besides offering a less technique-sensitive procedure.[2]

Good resin infiltration in a total-etched, wet-bonded specimen can be achieved if the adhesive resin replaces all the water within the demineralized matrix that was previously occupied by mineral, without collapse of the collagen matrix.[4] The techniques of etching, rinsing, application of adhesive resin, and evaporation of solvents in total-etch adhesives was simplified with the advent of self-etch adhesives.

Self-etching adhesive systems that are characterized by acidic monomers which are not rinsed from the tooth surface have become popular because their use has simplified a complicated technique. With the self-etch system fewer steps are required and there is no need for exercising any clinical judgment regarding the presence of residual dentinal moisture. These systems act by simultaneously conditioning, demineralizing, and infiltrating both enamel and dentin. The smear layer is altered but not removed, and rinsing is not indicated.[9]

The application of multiple layers and curing of successive layers using a self-etching primer system has been shown to increase bond strength.[4] It has been suggested that this technique eliminates the oxygen-inhibited zone at the top of the first coating of the bonding resin layer.[4] However, this method of multiple coatings without light curing between each layer increased the resin infiltration into the collagen web. That is, when the co-monomers are not polymerized, monomers can continue to diffuse inward, while solvents are diffusing outward. When multiple coats are applied but not cured, the resin infiltration of the hybrid layer and the removal of residual water may be more complete, without increasing the thickness of the overlying adhesive layer.[4] Although, the effectiveness of consecutive coats has been shown using a self-etching adhesive.[4] No similar report is available on the effect of multiple consecutive coatings of total-etch adhesives.

In the present study, the effect of single and multiple consecutive applications of total-etch adhesives and self-etch adhesives on tensile bond strength was evaluated. Tensile bond strength ultimately depends on resistance to tensile forces. With tensile bond strength tests it is possible to use a test specimen to compare regional adhesion on various dentin sites and the results have direct correlation with clinical situations. The highest bond strength was achieved following three consecutive applications of Gluma (total-etch) and two consecutive applications of AdheSE (self-etch). It is possible that the procedure of repeated adhesive application and the subsequent solvent evaporation may promote resin infiltration between the exposed collagen fibrils.[4]

Bond strength decreased when more than three consecutive multiple coatings of total-etch or two consecutive coats of self-etch were applied. This might be due to increase in the thickness of the adhesive layer, which acts as a weak interface. The increased resin infiltration into collagen caused by the consecutive coating method might remove residual water, thereby improving resin infiltration and cross-linking of the adhesive co-monomers within the hybrid layer.[4]

Total-etch technique (Gluma) gives higher bond strength than self-etch (AdheSE) because in total-etch, etching is followed by application of the bonding agent, which contains both primer and adhesive. On application of 35% phosphoric acid, the smear layer and superficial dentin are demineralized and the collagen fibers of superficially demineralized dentin are exposed. The exposed collagen may provide reactive groups that can chemically interact with bonding primers.[5] The ethanol solvent of Gluma, due to its high vapor pressure, competes with moisture and replaces it, promoting infiltration of monomer through the nano spaces of the exposed collagen network. This collagen network serves as a framework for the creation of a resin–demineralized dentin hybrid layer, resulting in a strong micromechanical interlocking between resin and the superficially demineralized dentin.[10]

Solvent evaporation can facilitate the polymerization reaction because solvent volatilization can reduce the distance among monomers and increase the degree of conversion. Acetone is frequently used as a solvent since it can efficiently remove water from surfaces. Ethanol is another organic solvent that is used as a vehicle in adhesives, but it has a higher boiling temperature and less high vapor pressure than acetone. Acetone does not expand dried demineralized dentin as alcohol does and it must be used only with the wet-bonding technique.[11] As the organic solvent evaporates, collapse of the collagen network is prevented because the collagen fibrils will be stiffened in the spongy expanded state. Moreover, alcohol based primer has a much higher vapor pressure which results in less surface tension on the collagen fibril network.[2] This could be the reason why higher bond strength can be achieved with total-etch adhesives than with self-etch adhesives.

In the self-etch technique (AdheSE), the primer contains a bis-acrylamide compound, which dissolves in water as well as in organic solvents. According to the manufacturer, the bis-acrylamide contains an amide and an acrylic group. The amide group binds with collagen and the acrylic with the monomer. The phosphoric acid in the self-etching primer creates channels through the smear layer and aids in the penetration of the primer, which coats the surface and binds the monomer. The hybrid layer produced by these systems are however usually thinner, with a limited resin-infiltrated dentin surface layer.[12]

Self-etching primers incorporate a significant amount of water as a solvent in order to promote the ionization of the acidic monomers. After solvent evaporation, the adhesive layer can be very thin and therefore the mechanical properties may be low. In addition, a demineralized dentin zone has been found below the hybrid layer formed by self-etching primers, which is not fully protected by the adhesive and this could jeopardize bond strength.[13]

The increase in bond strength obtained by increasing the number of consecutive coating of adhesives (up to three coats of total-etch and two coats of self-etch) suggests that this technique might be useful in all total-etch and self-etch systems.

## CONCLUSIONS

As per the conditions of the study it can be concluded that

1. The consecutive coating method with multiple applications of adhesive resin improves the quality of resin dentin bonds.
2. Total-etch gives higher bond strength compared to self-etch after multiple consecutive applications.
3. Three coats of total etch adhesive gives a better bond strength and two coats of self etch adhesive shows improved bond strength.
